# Effect of Omega-3 Polyunsaturated Fatty Acids Treatment on Lipid Pattern of HIV Patients: A Meta-Analysis of Randomized Clinical Trials

**DOI:** 10.3390/md18060292

**Published:** 2020-06-01

**Authors:** Federica Fogacci, Enrico Strocchi, Maddalena Veronesi, Claudio Borghi, Arrigo F. G. Cicero

**Affiliations:** 1 Atherosclerosis and Hypertension Research Group, Medical and Surgical Sciences Department, Sant’Orsola-Malpighi University Hospital, Building 2–IV Floor, Via Albertoni 15, 40138 Bologna, Italy; federicafogacci@gmail.com (F.F.); enrico.strocchi@unibo.it (E.S.); maddalena.veronesi@unibo.it (M.V.); claudio.borghi@unibo.it (C.B.); 2 Italian Nutraceutical Society (SINut), 40138 Bologna, Italy

**Keywords:** HIV, Omega-3 polyunsaturated fatty acids, triglycerides, high-density lipoprotein cholesterol, meta-analysis

## Abstract

Even though omega-3 polyunsaturated fatty acids (PUFAs) seem to be effective in the treatment of human immunodeficiency virus (HIV)-associated dyslipidemia, their impact is still debated. For this reason, our aim was to perform a meta-analysis of the clinical evidence available to date. A systematic literature search was conducted in order to identify published clinical trials assessing the effect of PUFAs treatment on serum lipoproteins, and its safety profile. The effect sizes for lipid changes were expressed as mean difference (MD) and 95% confidence interval (CI). For safety analysis, odd ratios and the 95% CI were calculated with the Mantel–Haenszel method. Data were pooled from nine clinical studies comprising overall 578 HIV-affected subjects. Meta-analysis of the data suggested that omega-3 PUFAs significantly reduced triglycerides (TG) (MD = −1.04, 95% CI: −1.5, −0.58 mmol/L, *p* < 0.001), while increasing high-density lipoprotein cholesterol (MD = 0.36, 95% CI: 0.12, 0.61 mmol/L, *p* = 0.004), without affecting serum levels of total cholesterol, very-low- and low-density lipoprotein cholesterol, and apolipoprotein B and A1. Change in TG was significantly associated with eicosapentaenoic acid administered via daily dose. PUFA treatment did not lead to an increased risk of adverse events. In conclusion, PUFAs are safe and exert a significant plasma lipid improving effect in HIV-positive patients.

## 1. Introduction

Among patients with chronic infection by human immunodeficiency virus (HIV), dyslipidemia is the most prevalent cardiovascular disease risk factor, being present in around 40% of the affected subjects [1]. The most common lipid alteration is hypertriglyceridemia, due both to HIV infection and the prevalence of several conditions (e.g., insulin resistance, hepatic steatosis and diabetes mellitus) leading to increased triglycerides (TG) [2]. Furthermore, elevated TG is a frequent side effect of antiretroviral treatment (ART) [3].

For the management of dyslipidemia in HIV-positive patients, the Infectious Disease Society of America (IDSA) and the Adult AIDS Clinical Trial Group (ACTG) refer to the updated recommendations from the National Cholesterol Education Program (NCEP) Expert Panel on Detection, Evaluation, and Treatment of High Blood Cholesterol in Adults (ATP III) [4,5].

A number of clinical trials have shown that in patients with chronic HIV infection, hypertriglyceridemia can be at least partially corrected by treatment with omega-3 polyunsaturated fatty acids (PUFAs) [6], with a lower risk of drug–drug interaction in comparison with fibrates [7,8].

Natural sources of omega-3 are found in both animal (fish, krill, egg, squid) and plant (algae, flaxseed, walnut, edible seeds, clary sage, seed) sources, in the form of docosahexaenoic (DHA) and eicosapentaenoic acid (EPA) or as alpha-linolenic acid (ALA) respectively [9].

The European Food Safety Agency (EFSA) established a health claim indicating that the intake of at least 2 g/day of DHA and EPA is able to maintain normal blood TG levels in the general population [9,10]. The American Heart Association (AHA) indicates doses ranging from 2 to 4 g/day of EPA and DHA to reduce TG levels by 25–30% [11]. However, the metabolic effect of PUFAs treatment in HIV-affected patients treated with ART is not yet clear. Consequently, we aimed to perform a meta-analysis on the clinical evidence available to date to better define its efficacy and tolerability profile.

## 2. Results

### 2.1. Flow and Characteristics of the Included Studies

**Figure 1:**
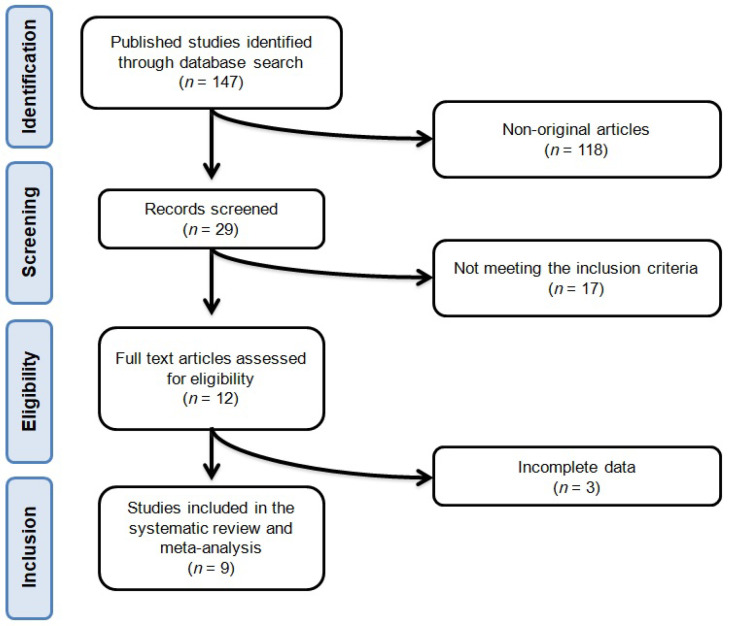
Flow chart of the number of studies identified and included in the meta-analysis.

After database searches performed strictly according to inclusion and exclusion criteria, 147 published articles were identified, and their abstracts reviewed. Of these, 118 were excluded because they were non-original articles. Another 17 were eliminated because they did not meet the inclusion criteria. Thus, 12 articles were carefully assessed and reviewed. An additional three studies were excluded because they reported incomplete data. Finally, nine studies were eligible and included in the meta-analysis [12,13,14,15,16,17,18,19,20]. The study selection process is shown in Figure 1.

Data were pooled from nine clinical trials comprising 18 treatment arms, which included 578 subjects, with 308 in the active-treated arm and 270 in the control one.

The eligible studies were published between 2006 and 2016. Follow-up periods ranged between 8 weeks and 6 months and different treatment regimens were tested. All selected trials were designed with parallel groups [12,13,14,15,16,17,18,19] or were crossover [20], and all were multicenter [13,15,17] or single-center [12,14,16,18,19,20] clinical studies. The enrolled subjects were adult patients living with chronic HIV infection and iatrogenic dyslipidemia. The baseline characteristics of the evaluated studies are summarized in Table 1.

### 2.2. Risk of Bias Assessment

According to the Cochrane criteria, almost all of the included studies were characterized by sufficient information regarding sequence generation, allocation concealment, and personal and outcome assessments. Some trials had a high risk of bias for incomplete outcome data and selective outcome reporting. Details of the quality of bias assessment are reported in Table 2.

### 2.3. Lipid-Lowering Effect of Omega-3 Polyunsaturated Fatty Acids

**Figure 2:**
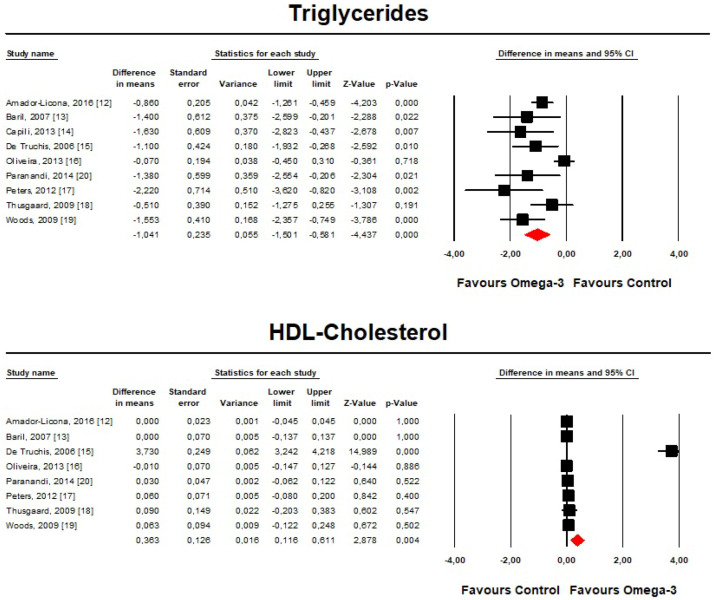
Forest plot displaying mean differences and 95% confidence intervals for the effect of treatment with omega-3 PUFA on plasma TG and HDL-C concentrations.

Meta-analysis of the data suggested that omega-3 PUFAs significantly reduced TG (mean difference (MD) = −1.04, 95% CI: −1.5, −0.58 mmol/L, *p* < 0.001; I^2^ = 69.3%) and high-density lipoprotein cholesterol (HDL-C) serum levels (MD = 0.36, 95% CI: 0.12, 0.61 mmol/L, *p* = 0.004; I^2^ = 96.9%) (Figure 2).

**Figure 3:**
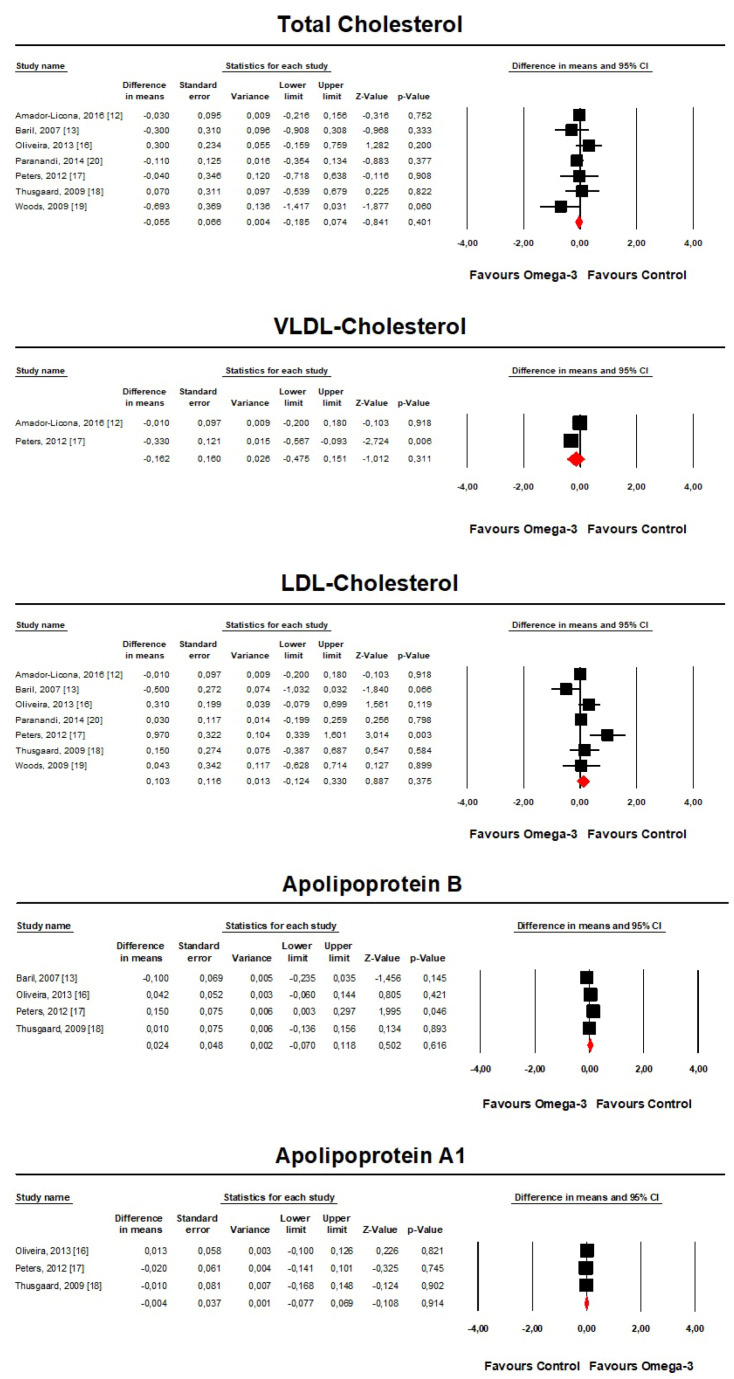
Forest plot displaying mean differences and 95% confidence intervals for the impact of treatment with omega-3 fatty acids on plasma TC, HDL-C and Apo-A1 concentrations.

However, the treatment did not exert any significant effect on total cholesterol (TC) (MD = −0.06, 95% CI: −0.19, 0.07 mmol/L, *p* = 0.401; I^2^ = 5.4%), very-low-density lipoprotein cholesterol (VLDL-C) (MD = −0.16, 95% CI: −0.48, 0.15 mmol/L, *p* = 0.311; I^2^ = 76.5%), low-density lipoprotein cholesterol (LDL-C) (MD = 0.1, 95% CI: −0.12, 0.33 mmol/L, *p* = 0.375; I^2^ = 58.7%), apolipoprotein B (Apo B) (MD = 0.02, 95% CI: −0.07, 0.12 mmol/L, *p* = 0.616; I^2^ = 51.8%) and apolipoprotein A-1 (Apo A-1) (MD = −0.004, 95% CI: −0.08, 0.07 mmol/L, *p* = 0.914; I^2^ = 0%) concentrations (Figure 3).

The effect sizes were robust in the leave-one-out sensitivity analysis and not mainly driven by a single study.

**Figure 4:**
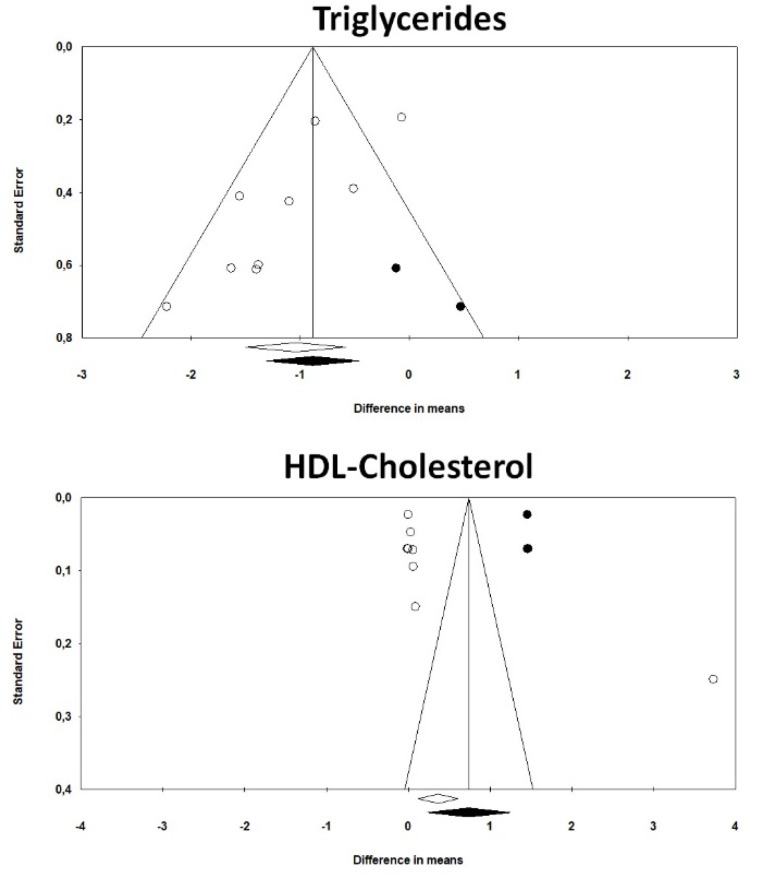
Funnel plots detailing publication biases in the studies included in the meta-analysis for the impact of treatment with omega-3 PUFAs on plasma TG and HDL-C concentrations.

Visual inspection of Begg’s funnel plots suggested potential publication biases for the effect of treatment with omega-3 PUFAs on serum TG and HDL-C concentrations. These observations were fully confirmed by Begg’s rank correlation (*p* = 0.01 in both cases) and partially confirmed by Egger’s regression asymmetry test (TG: *p* = 0.02; HDL-C: *p* > 0.5). The asymmetries were imputed to two potentially missing studies on the right side of the funnel plot which increased the estimated effect size on TG to −0.89 (95% CI: −1.31, −0.46) and three potentially missing studies on the same side of the funnel plot which increased the estimated effect size on HDL-C to 0.74 (95% CI: 0.23, 1.25) (Figure 4).

**Figure 5:**
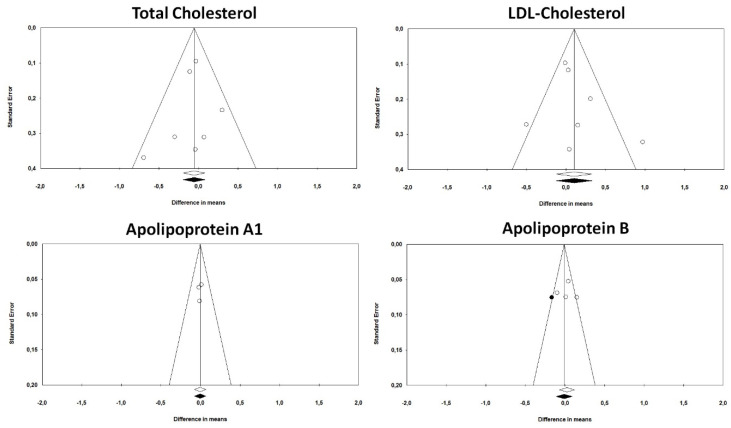
Funnel plots detailing publication biases in the studies included in the meta-analysis for the impact of treatment with omega-3 fatty acids on plasma TC, LDL-C, Apo-A1 and Apo-B concentrations.

The funnel plots of standard error by effect size (MD) for TC, LDL-C and Apo-A1 were symmetric, suggesting no publication bias for the outcomes (Figure 5). These observations were confirmed by Begg’s rank correlation and Egger’s regression test. However, visual inspection of Begg’s funnel plots suggested potential publication bias for Apo B concentrations. The asymmetry was imputed to one potentially missing study on the left side of the funnel plot reducing the estimated effect size to −0.01 (95% CI: −0.11, −0.09) (Figure 5). This observation was not confirmed by Begg’s rank-correlation method or Begg’s rank-correlation test (*p* > 0.5 in both cases).

Due to the inadequate number of studies on VLDL-C, publication bias tests were not applicable.

### 2.4. Differential Effectiveness of EPA and DHA on Lipids

**Figure 6:**
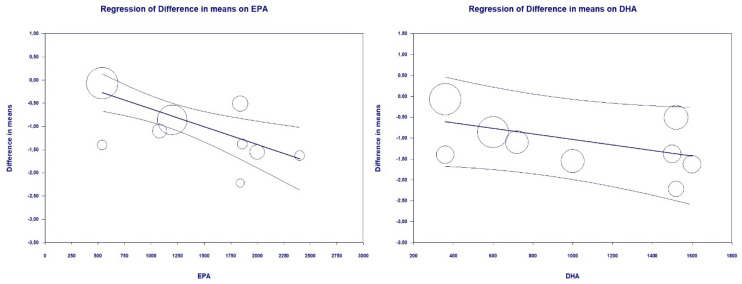
Meta-regression bubble plots of the association between mean difference in TG and treatment with EPA (**left**) and DHA (**right**). The size of each circle is inversely proportional to the variance of change.

Change in TG was significantly associated with EPA daily dose (slope= −0.0008, 95% CI: −0.0012, −0.0004, *p* < 0.001), although not with DHA daily dose (slope= −0.0007, 95% CI: −0.0014, 0.0001, *p* = 0.08) (Figure 6).

**Figure 7:**
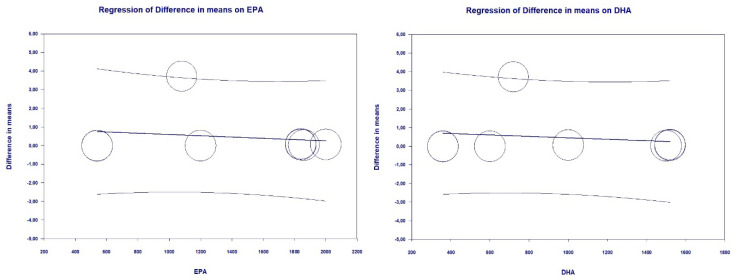
Meta-regression bubble plots of the association between mean difference in HDL-C and treatment with EPA (**left**) and DHA (**right**). The size of each circle is inversely proportional to the variance of change.

Treatment-dependent change in HDL-C was neither associated with EPA daily dose (slope = −0.0003, 95% CI: −0.0018, 0.0011, *p* = 0.64) nor with DHA daily dose (slope = −0.0004, 95% CI: −0.0021, 0.0013, *p* = 0.66) (Figure 7).

### 2.5. Safety Analysis for Omega-3 Fatty Acids Administration

The safety analysis included all the studies considered for the efficacy analysis, except for that by Peters et al. [17], which selectively reported the adverse events that occurred during the trial.

According to our analysis, the incidence of adverse events did not differ between groups (Table 3). The findings are robust in the leave-one-out sensitivity analyses.

## 3. Discussion

By analyzing data from nine clinical studies including 578 patients, this meta-analysis shows that omega-3 PUFAs significantly improve TG and HDL-C in patients with HIV chronic infection, with a favorable safety profile. The findings strengthen those previously reported by Oliveira and Rondò in a smaller sample of population [21], and emphasize the safety of PUFAs treatment in people with HIV with hypertriglyceridemia. This is of particular interest, since the pharmacological management of dyslipidemia associated with standard antiretroviral therapy (ART) or highly active retroviral treatment (HAART) is often complex for the risk of drug-drug interactions [22].

Even though lifestyle changes might improve the cardiometabolic risk of people with HIV, their efficacy is frequently limited and varies across settings [23]. Furthermore, iatrogenic hypertriglyceridemia increases the risk of acute pancreatitis in HIV-positive patients as well as in the general population [24]. In this event, the severity of hypertriglyceridemia varies, depending on the specific regimen (e.g., in stravudine-based HAART regimens TG were found to be higher than tenofovir) [25]. In such circumstances, TG-lowering pharmacological treatment should be considered [26].

Omega-3 PUFAs reduce TG synthesis through several mechanisms: reducing the amount of plasma fatty acids; increasing the synthesis of phospholipids; and finally decreasing the activity of TG-synthesizing enzymes (diacylgylcerol acyltranferase and phosphatidic acid phosphohydrolase) [27]. A recent meta-analysis of 86 randomized clinical trials (RCTs) including 162,796 participants showed that increasing PUFAs intake reduces plasma TG levels by 15% and slightly decreases the risk of coronary heart disease mortality (relative risk (RR) = 0.90, 95% CI: 0.81, 1.00) and coronary heart disease events (RR = 0.91, 95% CI: 0.85, 0.97) [28]. This is of particular interest in HIV-positive subjects, since the infection is associated with an increased risk of myocardial infarction compared with uninfected individuals (RR = 1.73; 95% CI: 1.44, 2.08), with HAART seeming to be a significant determinant of this risk [29]. Moreover, PUFAs could also have a positive impact on diseases other than cardiovascular ones [30], so our data support their use in HIV-positive patients. The effect of PUFAs on HDL-C is more debatable because it is strongly conditioned by the reduction in TG levels, which is dose-dependent [30]. From our data, it is also clear that the single study [15] where HDL-C plasma level was more significantly affected was also the one where an unusually high daily dose of PUFAs was tested.

The main limitation of this meta-analysis is related to the relatively small number of subjects involved in the trials, which were often short- or medium-term. The degree of heterogeneity for lipids change is another important limitation of the analysis. This could be partly related to another limitation of the included trials, where different formulations of PUFAs were tested. In fact, different EPA/DHA ratios and different pharmaceutical forms could be associated with variable effects on lipid pattern [31], because of the different impact of EPA and DHA on lipid fractions.

Notwithstanding these limitations, our data clearly indicate that treatment with omega-3 PUFAs in patients with chronic HIV infection is safe and effective in lowering TG and improving HDL-C serum levels.

## 4. Materials and Methods

The study was designed according to guidelines of the 2009 preferred reporting items for systematic reviews and meta-analysis (PRISMA) statement [32]. Due to the study design, neither Institutional Review Board (IRB) approval nor patient informed consent were required.

### 4.1. Search Strategy

PubMed, SCOPUS, Google Scholar and ISI Web of Science by Clarivate databases were searched, with no language restriction, using the following search terms: (“HIV” OR “Human Immunodeficiency Virus” OR “HIV+” OR “HIV-positive” OR “AIDS” OR “Acquired Immunodeficiency Syndrome”) AND (“Omega-3” OR “Omega 3” OR “PUFA” OR “Polyunsaturated fatty acids”) AND (“Lipids” OR “Lipid” OR “Lipid-lowering” OR “Total cholesterol” OR “TC” OR “Low-density lipoprotein cholesterol” OR “LDL-C” OR “LDL” OR “High-density lipoprotein cholesterol” OR “HDL-C” OR “HDL” OR “Triglycerides” OR “Triglyceride” OR “TG” OR “Apolipoprotein” OR “Apolipoprotein-B” OR “Apo-B” OR “Apo B” OR “Apolipoprotein-A1” OR “Apo-A1” OR “Apo A1”) AND (“Clinical trial” OR “Clinical study” OR “Pilot study”). The wild-card term “*” was used to increase the sensitivity of the search strategy, which was limited to studies on humans. The reference list of identified papers was manually checked for additional relevant articles. In particular, additional searches for potential trials included the references of review articles on the issue, and the abstracts from selected congresses on the subject of the meta-analysis. The literature was searched from inception to 25 April 2020.

All paper abstracts were screened by two reviewers (F.F. and E.S.) in an initial process to remove ineligible articles. The remaining articles were obtained in full-text and assessed again by the same two researchers, who evaluated each article independently and carried out data extraction and quality assessment. Disagreements were resolved by discussion with a third party (A.F.G.C.).

### 4.2. Study Selection Criteria

Original studies were included if they met the following criteria: (i) enrolling patients with HIV, (ii) being a clinical trial with either multicenter or single-center design, (iii) having an appropriate controlled design for treatment with omega-3 PUFAs and (iv) investigating the effect of omega-3 PUFAs on plasma lipids.

The exclusion criteria were: (i) lack of a control group for the intervention and (ii) lack of sufficient information about plasma lipids at baseline or follow-up. Studies were also excluded if they contained subjects that overlapped with other studies.

### 4.3. Data Extraction

The data abstracted from the eligible studies were: (i) first author’s name; (ii) year of publication; (iii) study design; (iv) follow-up; (v) main inclusion criteria; (vi) study groups; (vii) number of enrolled patients; (viii) sex and age of study participants; (ix) years since initial HIV diagnosis; (x) years since the start of ART therapy; and (xi) CD4+ T cell count at baseline. All data extraction and database typing were reviewed by the principal investigator (A.F.G.C.) before the final analysis, and doubts were resolved by mutual agreement among the authors.

### 4.4. Quality Assessment

A systematic assessment of the risk of bias in the included studies was performed using the Cochrane criteria [33]. The following items were used: adequacy of sequence generation, allocation concealment, blinding addressing of dropouts (incomplete outcome data), selective outcome reporting and other probable sources of bias [33]. Two reviewers (F.F. and E.S.) performed the risk-of-bias assessment independently and disagreements were resolved by a consensus-based discussion.

### 4.5. Data Synthesis

The meta-analysis was entirely conducted using Comprehensive Meta-Analysis (CMA) V3 software (Biostat, NJ) [34].

Net changes in the investigated parameters (change scores) were calculated by subtracting the value at baseline from the one after intervention, in the active-treated group and in the control group. Standard deviations (SDs) of the mean differences were obtained as follows, reported by Follman and colleagues: SD = √[SD_pre_^2^ + SD_post_^2^ − (2R × SD_pre_ × SD_post_)], assuming a correlation coefficient (R) = 0.5 [35]. If the outcome measures were reported as median and range (or 95% CI), the mean and SD values were estimated using the method described by Wan et al. [36]. The studies’ findings were combined using a fixed-effect model or a random-effect model (using the DerSimonian–Laird method) and the generic inverse variance method based on the level of inter-study heterogeneity, which was quantitatively assessed using the Higgins index (I^2^) [37]. Effect sizes for changes in lipids were expressed as MD and 95% CI. For safety analysis, OR and 95% CI intervals were calculated using the Mantel–Haenszel method [38]. Safety analysis was performed by excluding studies with zero events in both arms. If one or more outcomes could not be extracted from a study, the study was removed from the analysis involving those outcomes only. Adverse events were considered in the analysis only if occurring in at least two of the included clinical trials.

Sensitivity analysis was conducted using the leave-one-out method (i.e., removing one study at a time and repeating the analysis) in order to evaluate the influence of each single study on the overall observed effect size [39].

The EPA and DHA daily administered doses were sequentially entered into a random-effect meta-regression model to explore their association with the estimated effect sizes.

Two-sided *p*-values ≤ 0.05 were considered as statistically significant for all tests.

### 4.6. Publication Biases

Potential publication biases were explored using visual inspection of Begg’s funnel plot asymmetry, Begg’s rank correlation test, and Egger’s weighted regression test [40,41]. The Duval and Tweedie “trim and fill” method was used to adjust the analysis for the effects of publication biases [42]. Two-sided *p*-values ≤ 0.05 were considered statistically significant.

## 5. Conclusions

In conclusion, based on the results of this meta-analysis of randomized clinical studies, treatment with omega-3 PUFAs seems to exert a favorable effect on TG and HDL-C serum levels, being suggestive of a positive prognostic effect. Further clinical trials are expected to investigate the long-term safety of the treatment.

## Figures and Tables

**Table 1:** Baseline characteristics of the included studies.

First Author, Year	Study Design	Follow-Up	Main Inclusion Criteria	Study Group	Patients (n)	Male (n (%))	Age (Years; Mean ± SD)	Years Since HIV Diagnosis (Mean ± SD)	Years Since ART Therapy Started (Mean ± SD)	CD4+ T Cell Count (cell/mL)
Amador-Licona, 2016 [12]	Randomized, double-blind, placebo-controlled, parallel-group, clinical study	6 months	HIV infection treated with stable HAART regimen for ≥ 3 months; TG ≥ 2.26 mmol/L and ≤ 5.65 mmol/L; LDL-C ≥ 3.36 mmol/L and ≤ 4.13 mmol/L; CD4+ T cell count> 200 cell/mL	2.4 g/day omega-3 PUFA (EPA/DHA 1200/600 mg/day)	35	28 (80)	39.9 ± 9.5	5.6 ± 2	4.5 ± 1.7	525.7 ± 129.6
				Placebo	30	23 (65.7)	39.9 ± 8	6.8 ± 2.2	5.4 ± 2	663.7 ± 180
Baril, 2007 [13]	Multicenter, randomized, open-label, placebo-controlled, parallel-group, clinical study	12 weeks	HIV infection treated with stable ARV regimen for ≥6 months; TG ≥ 6 mmol/L and ≤ 6 mmol/L	3 g/day salmon oil omega-3 PUFA (EPA/DHA 540/360 mg/day)	26	26 (100)	50.9 ± 8.4	9.9 ± 5.2	NA	736 ± 456
				Placebo	32	31 (96.9)	47.8 ± 5.5	11.8 ± 5.2	NA	540 ± 307
Capili, 2013 [14]	Randomized, double-blind, placebo-controlled, parallel-group, clinical study	8 weeks	HIV infection treated with stable PI-ART regimen for ≥ 6 months; TG ≥ 1.69 mmol/L and ≤ 5.65 mmol/L; LDL-C < 3.36 mmol/L; CD4+ T cell count ≥ 300 cells/mL	4 g/day omega-3 PUFA (EPA/DHA 2400/1600 mg/day)	8	6 (75)	46.9 ± 11.5	9.5 ± 6.1	NA	573 ± 284
				Placebo	10	6 (60)	45.6 ± 6.5	12.6 ± 4.9	NA	525 ± 182
De Truchis, 2006 [15]	Multicenter, randomized, double-blind, placebo-controlled, parallel-group, clinical study	8 weeks	HIV infection treated with stable HAART regimen for ≥ 2 months; TG ≥ 3.43 mmol/L	6 g/day omega-3 PUFA (EPA/DHA 1080/720 mg/day)	58	52 (89.7)	45.6 ± 8.6	11 ± 4.5	7.1 ± 2.8	NA
				Placebo	62	55 (88.7)	47.1 ± 8.4	11.6 ± 4.2	7.7 ± 3.1	NA
Oliveira, 2013 [16]	Randomized, double-blind, placebo-controlled, parallel-group, clinical study	24 weeks	HIV infection treated with stable ART regimen for ≥ 3 months; TG > 1.3 mmol/L; LDL-C < 4.14 mmol/L; FPG < 7 mmol/L	3 g/day omega-3 PUFA (EPA/DHA 540/360 mg/day)	63	33 (76.7)	43.1 ± 7.4	10.3 ± 5.7	8.3 ± 4.1	591.8 ± 259.6
				Placebo	40	31 (77.5)	42.8 ± 6.3	10.9 ± 5	9.2 ± 3.5	616.2 ± 366.9
Paranandi, 2014 [20]	Randomized, double-blind, placebo controlled, crossover, clinical study	12 weeks	HIV infection treated with stable HAART regimen for ≥ 3 months; TG ≥ 1.69 mmol/L	4 g/day omega-3 PUFA (EPA/DHA 1860/1500 mg/day)	41	35 (85)	51.7 ± 9.6	16.7 ± 5.2	NA	621.3 ± 277
				Placebo	20					
Peters, 2012 [17]	Multicenter, randomized, double-blind, placebo-controlled, parallel-group, pilot clinical study	12 weeks	HIV infection treated with stable HAART regimen for ≥ 3 months; TG ≥ 3.39 mmol/L and ≤ 11.3 mmol/L; lipid-lowering treatment with fibrate or niacin	4 g/day omega-3 PUFA (EPA/DHA 1840/1520 mg/day)	23	23 (100)	46.1 ± 2.9	NA	NA	633 ± 217
				Placebo	25	24 (96)	43.6 ± 8.9	NA	NA	546 ± 257
Thusgaard, 2009 [18]	Randomized, double-blind, placebo-controlled, parallel-group, clinical study	12 weeks	HIV infection treated with stable ART regimen for ≥ 3 months	3.6 g/day omega-3 PUFA (EPA/DHA 1840/1520 mg/day)	26	19 (73)	43 ± 10	NA	8.1	503 ± 306
				Placebo	25	21 (84)	47 ± 11	NA	8	483 ± 267
Woods, 2009 [19]	Randomized, open label, diet-controlled, parallel-group, clinical study	10 weeks	HIV infection; TG > 1.69 mmol/L and/or QUICKI score < 0.35 or > 0.30	3 g/day omega-3 PUFA (EPA/DHA 2000/1000 mg/day)	28	24 (86)	46.2 ± 8.2	NA	NA	527.3 ± 225.2
				Control diet	26	19 (73)	46.3 ± 5	NA	NA	489.7 ± 228.1

ART = antiretroviral treatment; ARV = antiretroviral; DHA = docosahexaenoic acid; EPA = eicosapentaenoic acid; FPG = fasting plasma glucose; HAART = highly active antiretroviral therapy; HIV = human immunodeficiency virus; LDL-C = low-density lipoprotein cholesterol; NA = not available; PI-ART= Protease inhibitor based antiretroviral therapy; PUFA = polyunsaturated fatty acids; QUICKI = quantitative insulin sensitivity check index; SD = standard deviation; TG = triglycerides.

**Table 2:** Quality of bias assessment of the included studies according to Cochrane guidelines.

Author, Year	Sequence Generation	Allocation Concealment	Blinding of Participants, Personnel and Outcome Assessment	Incomplete Outcome Data	Selective Outcome Reporting	Other Potential Threats to Validity
Amador-Licona, 2016 [12]	L	L	L	L	L	L
Baril, 2007 [13]	L	L	L	H	L	H
Capili, 2013 [14]	L	L	L	H	H	U
De Truchis, 2006 [15]	L	L	L	H	H	U
Oliveira, 2013 [16]	L	L	U	L	L	L
Paranandi, 2014 [20]	U	U	L	L	L	L
Peters, 2012 [17]	L	L	L	L	L	H
Thusgaard, 2009 [18]	L	L	L	L	L	L
Woods, 2009 [19]	U	U	H	L	U	U

L = low risk of bias; H = high risk of bias; U = unclear risk of bias.

**Table 3:** Adverse events that occurred in at least two clinical trials.

Adverse Event	Number of Studies	Odd Ratio	95% Confidence Interval		Z-Value	*p*-Value	I^2^
			Lower Limit	Upper Limit			
Renal colic and urinary stones	2	5.34	0.61	46.59	1.517	0.129	0%
Nausea	2	4.33	0.47	40.4	1.287	0.198	0%
Flatulence	4	3.47	0.88	13.63	1.781	0.075	0%
Diarrhea	5	2.3	0.79	6.72	1.528	0.127	0%
Generic gastrointestinal disorders	3	1.25	0.55	2.82	0.534	0.593	0%
Cholelithiasis	2	1.04	0.11	10.33	0.036	0.971	1%
Skin rash	2	1.02	0.1	10.2	0.015	0.988	0%
Heartburn	2	1	0.14	7.02	0.001	0.999	0%
Generic infections	2	0.67	0.3	1.48	−0.989	0.322	0%
